# Consider the Source: Adolescents and Adults Similarly Follow Older Adult Advice More than Peer Advice

**DOI:** 10.1371/journal.pone.0128047

**Published:** 2015-06-01

**Authors:** Frederico S. Lourenco, Johannes H. Decker, Gloria A. Pedersen, Danielle V. Dellarco, B. J. Casey, Catherine A. Hartley

**Affiliations:** Sackler Institute for Developmental Psychobiology, Weill Cornell Medical College, New York, NY, United States of America; The University of Kansas Medical Center, UNITED STATES

## Abstract

Individuals learn which of their actions are likely to be rewarded through trial and error. This form of learning is critical for adapting to new situations, which adolescents frequently encounter. Adolescents are also greatly influenced by their peers. The current study tested the extent to which adolescents rely on peer advice to guide their actions. Adolescent and young adult participants completed a probabilistic learning task in which they chose between four pairs of stimuli with different reinforcement probabilities, with one stimulus in each pair more frequently rewarded. Participants received advice about two of these pairs, once from a similarly aged peer and once from an older adult. Crucially, this advice was inaccurate, enabling the dissociation between experience-based and instruction-based learning. Adolescents and adults learned equally well from experience and no age group difference was evident in the overall influence of advice on choices. Surprisingly, when considering the source of advice, there was no evident influence of peer advice on adolescent choices. However, both adolescents and adults were biased toward choosing the stimulus recommended by the older adult. Contrary to conventional wisdom, these data suggest that adolescents may prioritize the advice of older adults over that of peers in certain decision-making contexts.

## Introduction

Individuals readily learn through trial and error which actions are likely to be rewarded, and adolescents are no exception. Altering behavior in response to positive or negative outcomes is a critical process through which one learns to take beneficial actions. This capacity to recruit past experience to adjust one’s behavior improves across development [1], enabling adaption to the new situations frequently faced by adolescents [2–5]. Individuals also readily learn from the examples and advice of those around them. Learning through instruction can be more efficient, a fact recognized by parents who attempt to prevent their children from encountering potentially dangerous situations. As adolescents begin to spend more time with their peers and less time with their parents, they face the challenge of negotiating social interactions and novel decisions without this parental guidance [6]. Strikingly, one in five parents believe they have little influence over their teen’s choices, especially in the context of risky behaviors [7]. In contrast, there is mounting evidence for a heightened influence of peers on adolescent behavior [8,9]. As adolescents become less dependent on family support [6], they show increasing sensitivity to peers [10–12] and social cues [13,14], and less conformity to parental expectations [15,16].

Decision-making theories suggest that individuals make choices based largely on the subjective value (social, financial, hedonic) of available options [17]. Adolescents have been suggested to have enhanced value representations of potential outcomes, whether monetary [18–22] or social [23]. As such, both social contexts and desire for peer acceptance become key factors that influence adolescents’ decisions and actions [24]. This increased sensitivity to peer influence during adolescence has been associated with poor choices [8,9], such as reckless driving [25], substance abuse [26], and criminal behavior [27]. One potential factor underlying this heightened peer influence might be an increased weight placed on advice from peers versus that of parents, or other older adults. However to date, there has been little empirical research assessing the extent to which adolescents preferentially rely on advice from different sources.

Recent work in adults has suggested that advice influences behavior by biasing how positive and negative feedback gets processed [28–31]. Advice acts to create a “confirmation bias”, such that outcomes consistent with that advice influence future behavior more than inconsistent outcomes [31–33]. Previous work examining the influence of explicit instruction on learning has predominantly used inaccurate advice, in which the resulting bias leads to suboptimal decisions [29,34]. Performance on simple feedback-based learning tasks often approaches ceiling levels through experience alone. By placing the information gathered through experience in direct competition with advice, inaccurate instruction enables clear dissociation of the degree to which future actions are influenced by each form of information [28,29]. Previous studies using this task design have shown strong evidence of the biasing influence of advice in adults [28,29,32,34]. In contrast, a recent study examining the influence of explicit instruction on reward learning across development showed that children and adolescents did not exhibit this biasing effect [31]. However, in this study, the advice was simply displayed on the screen, devoid of any social source or significance. Here we modified the task, providing adolescents and young adults with advice from a similarly aged peer as well as an older adult. As adolescents often exhibit heightened sensitivity to peers [9,12], we hypothesized that adolescents’ choices might be biased by advice from a peer, whereas they might be less influenced by advice from an older adult.

## Materials and Methods

### Participants

A total of 54 volunteers participated in the experiment. Two adolescent males did not believe the advice manipulation, as assessed during debriefing, and were excluded. Forty-five participants—20 adolescents (age range = 12–17, *M* = 14.8, *SD* = 2.0) and 25 adults (age range = 18–30, *M* = 22.9, *SD* = 3.6)—completed the test phase with performance above chance for the pairings of the three 70% stimuli versus the 30% uninstructed stimulus. Five adolescents (3 males, 2 females) and 2 female adults were excluded for below chance test phase performance on these pairs. Adolescents had Tanner pubertal stages [35] of 2 or higher, including four (3 female) 12 year-olds. All participants reported no history of neurological disorders or color blindness. All participants and the parents of all minors (aged 12–17 years) provided written informed consent prior to testing, were debriefed, and received compensation according to the protocol approved by the Institutional Review Board (IRB) of Weill Cornell Medical College.

### Intelligence assessment

Thirty-nine (19 adolescents and 20 adults) of the 45 participants completed the Wechsler Abbreviated Scale of Intelligence [36] in order to obtain an intelligence quotient (IQ) estimate. There was no IQ difference between age groups (adolescents: M = 105.7, SD = 13.8; adults: M = 108.5, SD = 11.8; t(37) = -0.45, p = 0.66).

### Task procedure

**Fig 1:**
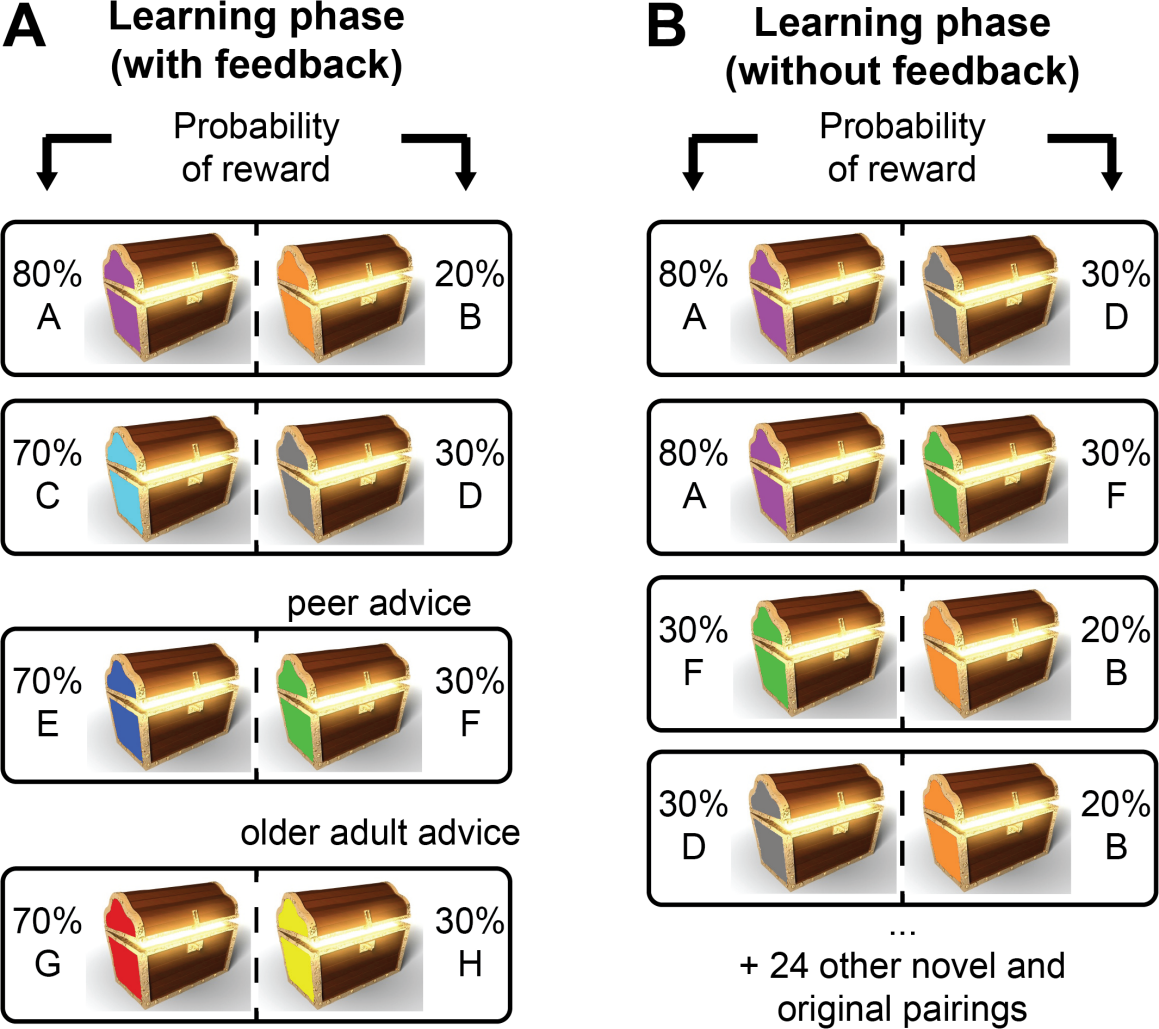
Task design and advice manipulation. **(**A) Learning phase: participants were presented with 60 trials of each stimulus pair (AB, CD, EF and GH), pseudo-randomized within 10-trial blocks and could learn through feedback the reward value of the stimuli. A peer and an older adult, both alleged to be previous participants, recommended choosing a suboptimal stimulus (“choose F” and “choose H,” respectively) (B) Test phase: participants were presented with all possible stimulus combinations (28 in total, 4 previously learned pairs and 24 novel pairs) without choice feedback, enabling the assessment of the learned value associated with each stimulus.

Participants completed an adapted probabilistic learning task [28,29], consisting of a learning phase and subsequent test phase (Fig 1). During learning, participants saw one of four stimulus pairs per trial (AB, CD, EF and GH), which were displayed as distinct colored treasure chests. Following each stimulus choice, feedback indicated whether it was “correct” or “incorrect”. For pair AB, choosing “A” led to positive feedback in 80% of trials, while choosing “B” was rewarded on only 20% of trials. The other three pairs (CD, EF, GH) had a 70/30% reward probability structure. For two pairs (EF and GH), participants were given incorrect advice—specifically, the less frequently rewarded stimulus was recommended. Importantly, for one pair (EF) the advice came from a peer and for the other pair (GH) the advice came from an older adult (Fig 1A), but it was inaccurate in both cases. Incorrect advice allows for clearer dissociation of whether choices were based on the experienced feedback or the received advice [29,34]. We manipulated the source of advice in order to reveal any differential influence of a peer or older adult advisor.

In the learning phase, the 240 trials (60 per pair) were presented for 2 seconds each. In the test phase, 168 trials (6 per pair) were presented with no time limit. Trial presentation was pseudo-randomized to ensure an equal number of presentations of each pair across blocks of trials. Stimulus color was counterbalanced between participants and left-right screen display was counterbalanced within participants across trials. For each pair, participants were asked to determine which chests were better and to accumulate tokens (“money bags”), which would be converted to cash compensation at the end of the experiment. In the learning phase, participants received feedback, thus they could learn through experience that the advice provided by the peer and the older adult was inaccurate. In the test phase, participants were presented with all 28 possible pair combinations, without feedback. They were asked to select the stimulus most likely to be correct and to “follow their gut” when in doubt. This enabled us to gauge the degree to which individuals learned the true stimulus values through experience alone, as well as the extent to which inaccurate advice from either source biased this trial-and-error learning. All participants received equal compensation, including a “performance-based bonus”, consistent with the IRB approved protocol.

### Manipulation of advice source

Participants received advice from two alleged previous participants: a gender- and approximate age-matched peer, and a gender-matched older adult. The same older adult advisors were used for participants in both age groups and were significantly older than participants (estimated by both groups to be approximately 43 years old). The adolescent group estimated their peer to be approximately 17.7 years old, whereas as the young adults estimated their peer to be approximately 26.9 years old. Through the use of vignettes (a picture of each advisor, an accompanying written message, and an image of the recommended chest), participants were advised to select two stimuli with low (30%) reward probabilities (chests F and H, Fig 1A). The peer advisor suggested that (*verbatim*) “it was difficult at first to figure out which chest was the best, but I’m sure it was this one”, while the older adult advised that “this was a very interesting task, a really good workout for your memory; I’m sure this chest was the best one”. To promote the believability of advisors, participants were asked prior to the task if they would be willing to provide advice to future participants by identifying their preferred chest and having their picture taken. All but four participants (1 adolescent, 3 adults) explicitly recalled both peer and older adult advice during debriefing.

### Data Analysis

Forty-five participants with test phase performance above chance for the 70/30% uninstructed pairs (CD, ED, GD) were included in our analyses. We analyzed learning phase data using a generalized linear mixed-effects model, based on the lme4 package for the R-statistics language [37], as described previously [31]. Optimal choice (i.e., for each pair, choosing the stimulus with the highest reward probability) was modeled with independent predictors of age group (adolescents, adults), pair (AB, CD, EF, GH), trial (1:240, z-normalized), and their interactions. Because of task complexity, IQ was treated as a covariate in all analyses. Missing IQ estimates for 6 participants (1 adolescent and 5 adults) were replaced with the mean IQ of their respective age group. We used a maximal random-effects structure [38], including per-participant adjustments to the intercept, pair, trial, and pair-by-trial interaction terms, as well as all possible random correlation terms among the random effects. We calculated p-values and 95% confidence intervals of the log-odds estimates through bootstrapping (bootMer function, 200 simulations, lme4 package), while p-values for analyses of variance were determined using likelihood-ratio-tests (mixed function, afex package).

**Fig 2:**
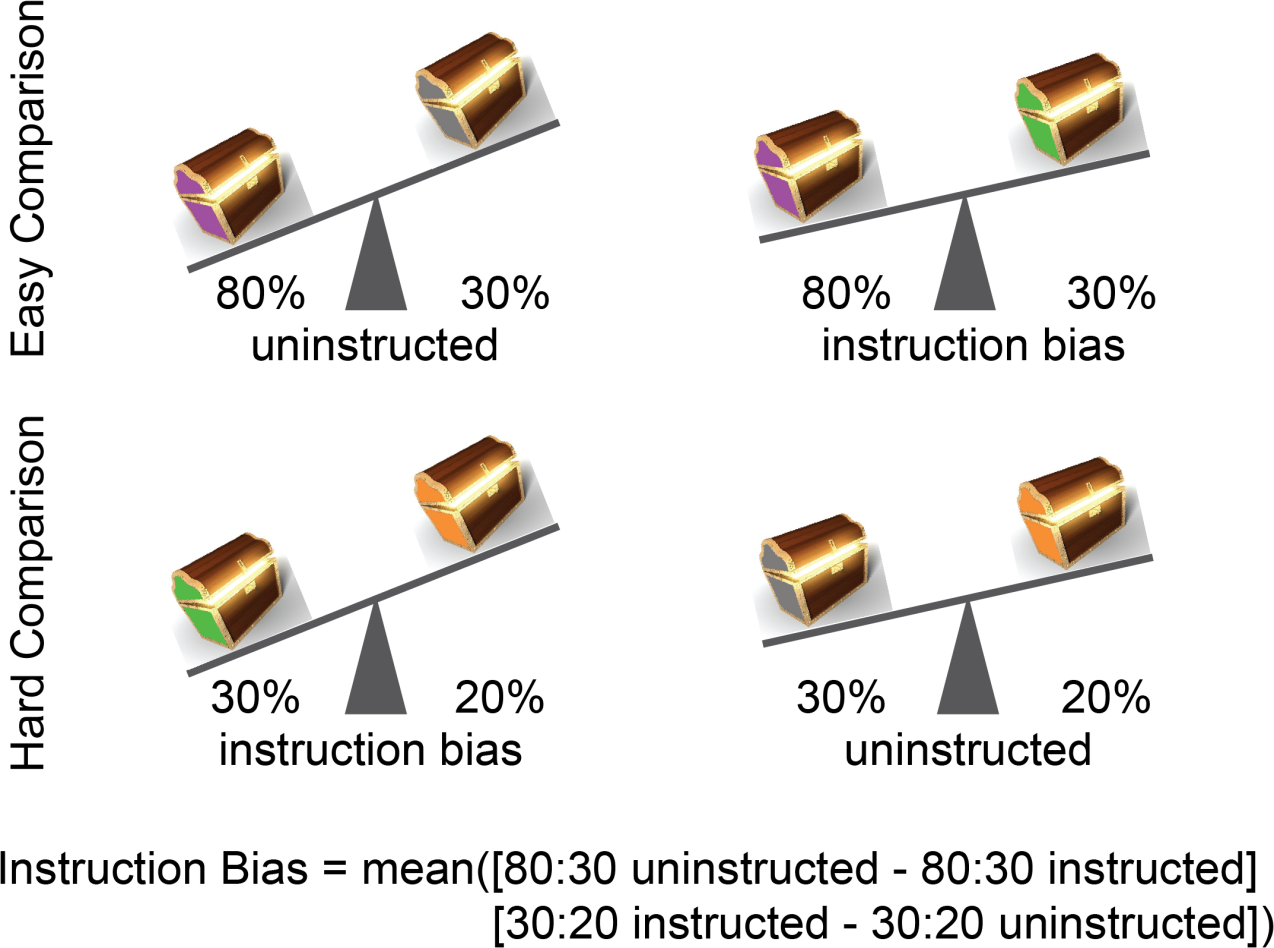
Peer and older adult instruction biases. Instruction bias scores were calculated by comparing performance on equally valued but differentially instructed pairs, averaging an easier (80% versus 30%) and harder (30% versus 20%) comparison. Peer and older adult instruction bias scores quantified the extent to which either source of advice biased participants’ baseline tendency to choose the higher valued option.

The influence of instruction was determined by examining test phase choices for equally valued but differentially instructed novel pairs, for which participants could not rely on any choice heuristics that may have developed for the pairs presented during the learning phase. First, we assessed whether participants favored the recommended stimulus when the stimulus pair reward probabilities were equal (DF and DH pairs, both 30/30% instructed). Next, the biasing effects of the peer and older-adult advice were quantified by calculating an instruction bias score [31] from a subset of test phase choice pairs that included the instructed or uninstructed 30% stimuli (an easier comparison: 80% versus 30% and a harder comparison 30% versus 20%, Fig 2). Performance was measured as the proportion of times a subject made the optimal choice. An instruction bias means that a participant chooses the instructed 30% stimulus more than the corresponding uninstructed 30% stimulus. This means for the easy (80:30) pair, where choosing the 80% stimulus is optimal, instruction consistent bias impairs performance relative to the uninstructed 80:30 pair. However, for the hard (30:20) pair, where choosing the 30% stimulus is optimal, a bias will improve performance. The instruction bias score averages these measures of improved and hindered performance because they reflect the same tendency to overvalue the instructed stimulus [31]. Positive scores (max = 1) indicate an instruction-consistent bias, while negative scores (min = -1) indicate an instruction-inconsistent bias (a tendency to go against advice). The peer and adult instruction bias scores were calculated identically incorporating the appropriate 30% instructed stimulus (H or F) into the following formula: Bias = mean([80:30 uninstructed]–[80: 30 instructed] + [30 instructed: 20]–[30 uninstructed: 20]). This instruction bias was also more rigorously assessed by analyzing choice behavior for the pairs underlying these bias scores using a generalized linear mixed-effects model with independent variables of age group (adolescents, adults), pair (easy 80/30%, hard 30/20%), and instruction (uninstructed, peer instructed, adult instructed). Finally, to determine whether participants demonstrated above chance experiential learning, we tested performance on all 6 uninstructed pairs (A, B, C, D combinations).

## Results

### Learning Phase Performance

**Fig 3:**
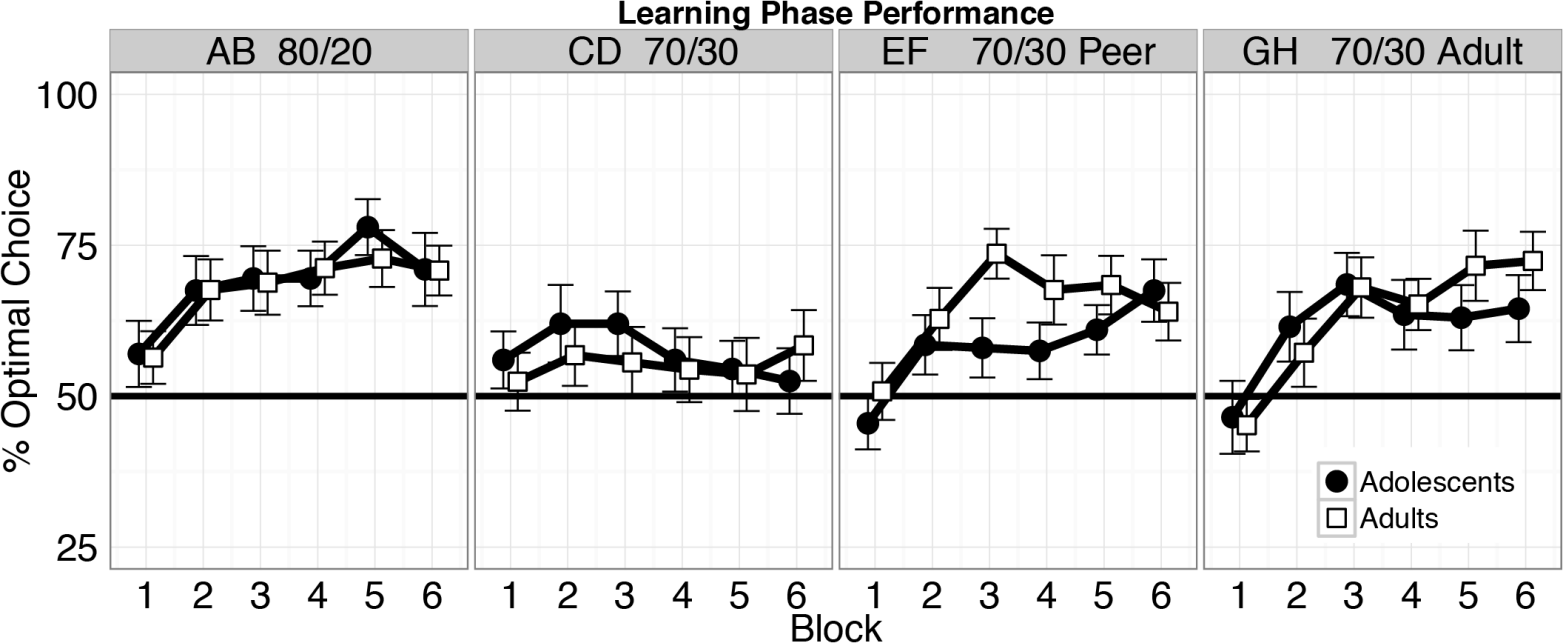
Learning phase performance. Choice performance was measured as the percentage of trials in which participants selected the most frequently rewarded stimulus of each pair (% optimal choice), presented in 10-trial blocks. Both adolescents (black) and adults (white) progressively learned the estimated value of the stimuli; by the end of the learning phase, they were significantly better than chance at choosing the optimal stimuli. Error bars represent SEM.

The learning phase was examined by testing for differences in performance, measured as making the optimal choice, by age group, pair, and trial. We found no difference in overall performance between age groups (p = 0.68; Fig 3). There was a main effect of stimulus pair (*Χ* ^2^ = 10.97, df = 3, p = 0.012), such that performance for the easier uninstructed (AB 80:20) pair was better than the harder uninstructed pair (CD 70:30; difference = 0.66, CI (0.26,1.06), p = 0.02) and the peer instructed pair (EF 70:30; difference = 0.46, CI (0.13,0.79), p = 0.01), but not the adult instructed pair (GH 70:30; p = 0.18). However, there was no difference between the instructed pairs (p = 0.28). Thus, performance was best for the easier uninstructed (80:20) and adult instructed pairs. Participants of both age groups exhibited similar performance on all pairs (age-group-by-pair: p = 0.83).

As expected, task performance improved throughout the learning phase for all participants (trial effect, *Χ* ^2^ = 27.35, df = 1, p <. 0001), with no age group differences (group-by-trial interaction: p = 0.33). Performance improved at different rates depending on the stimulus pair (*Χ* ^2^ = 11.18, df = 3, p = 0.01). Performance for the easier uninstructed pair (80:20) improved faster than the harder uninstructed pair (70:30; difference = 0.87, CI (0.41,1.34), p <. 001), but was similar to that of the peer (p = 0.08) and adult instructed pairs (p = 0.97). Participants of both age groups learned at similar rates across all pairs (age-group-by-pair-by-trial interaction effect: p = 0.35). Overall performance on the last block of trials was better than chance (t(44) = 6.98, p <. 0001), with no age group differences (F_1,43_ = 0.32, p = 0.57). Thus, despite receiving inaccurate advice, participants learned to choose the higher-valued stimulus through trial and error.

### Test Phase Performance

Overall performance for the 22 pairs for which there was an optimal choice (i.e., excluding 30:30 and 70:70 pairs) was better than chance (t(44) = 9.01, p <. 0001) and did not differ by age group (F_1,43_ = 0.21, p = 0.65). Likewise, performance for the 6 uninstructed pairs (A, B, C, and D combinations) was better than chance (estimate = 0.75, CI (0.55, 0.95), p = 0.03), and did not differ by age group (p = 0.53).

**Fig 4:**
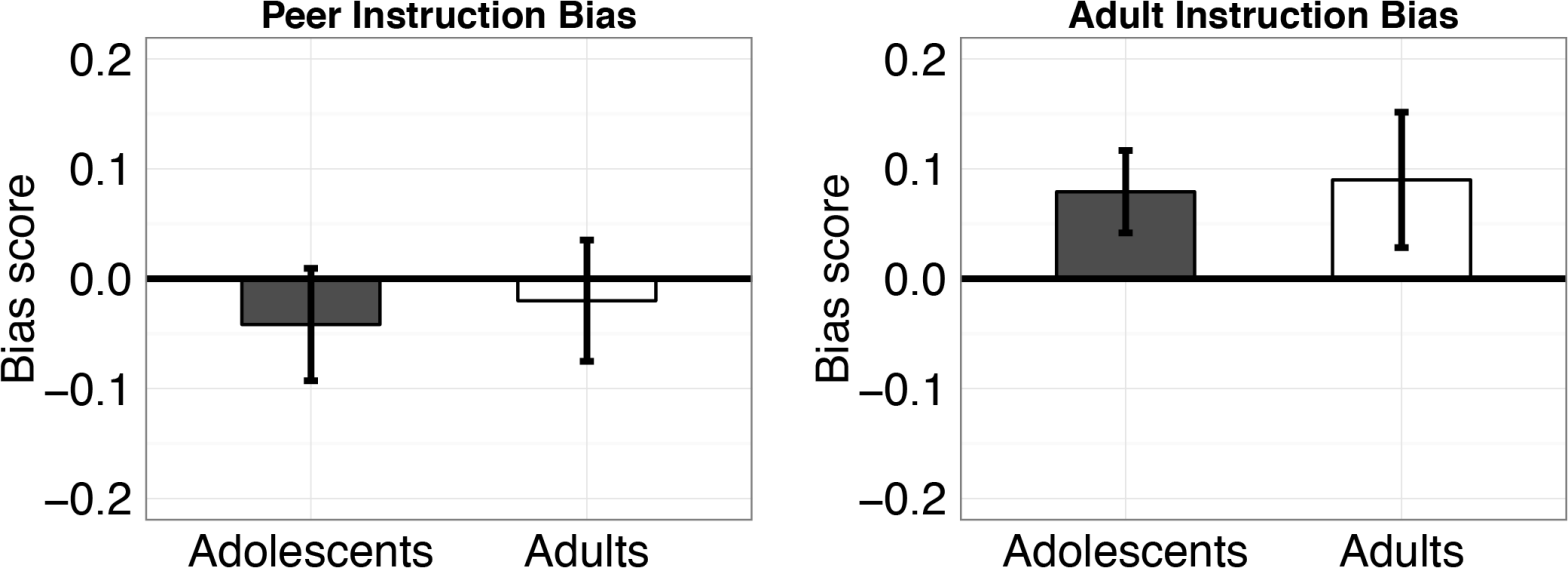
Test phase advice preference. For equally rewarded stimuli (D, F and H had a 30% reward probability), both age groups show a preference for the adult recommended (H), but not the peer recommended (F) stimulus, when compared to the uninstructed (D) stimulus. Error bars represent SEM.

First, we examined the choice preferences between pairings of the three differentially instructed 30% rewarded stimuli (Fig 4). Participants chose the adult recommended stimulus (H; t(44) = 3.35, p = 0.002), but not the peer recommended stimulus (F: t(44) = -1.04, p = 0.31), significantly more than the uninstructed stimulus (D). There was no preference for the adult recommended stimulus (H) over the peer recommended stimulus (F) (t(44) = 1.63, p = 0.109). There were no age group differences when comparing the source of advice (all p-values > 0.35).

**Fig 5:**
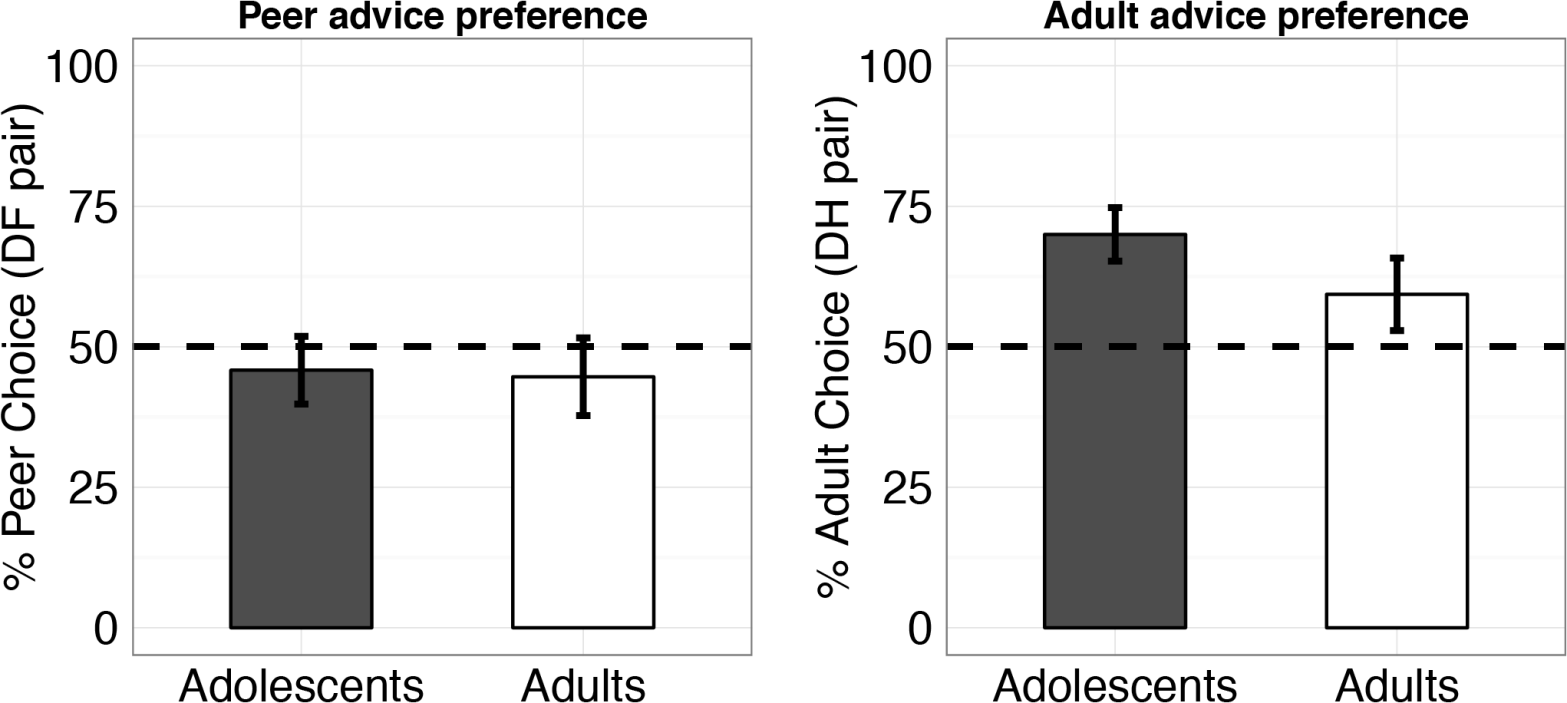
Test phase instruction biases. Older adult advice biased both adolescents and adults, while neither group were biased by peer advice. Error bars represent SEM.

Next, we examined the instruction bias scores, which compare performance on a broader set of equally valued but differentially instructed pairs. Any difference in performance can be attributed to a biasing effect of instruction. The average instruction bias score across both sources was not significantly different from zero (t(44) = 0.87, p = 0.39), suggesting that advice by itself does not induce a bias. However, when considering the source of advice (Fig 5), we found an older adult instruction bias (difference = 0.08, CI = (0.01, 0.16), t(44) = 2.26, p = 0.029), but no peer instruction bias (t(44) = -0.78, p = 0.44). The older adult bias was significantly larger than the peer bias (t(44) = 2.88, p = 0.006), and there was no difference between age groups in the biasing influence of older adult (p = 0.88) or peer advice (p = 0.77).

**Fig 6:**
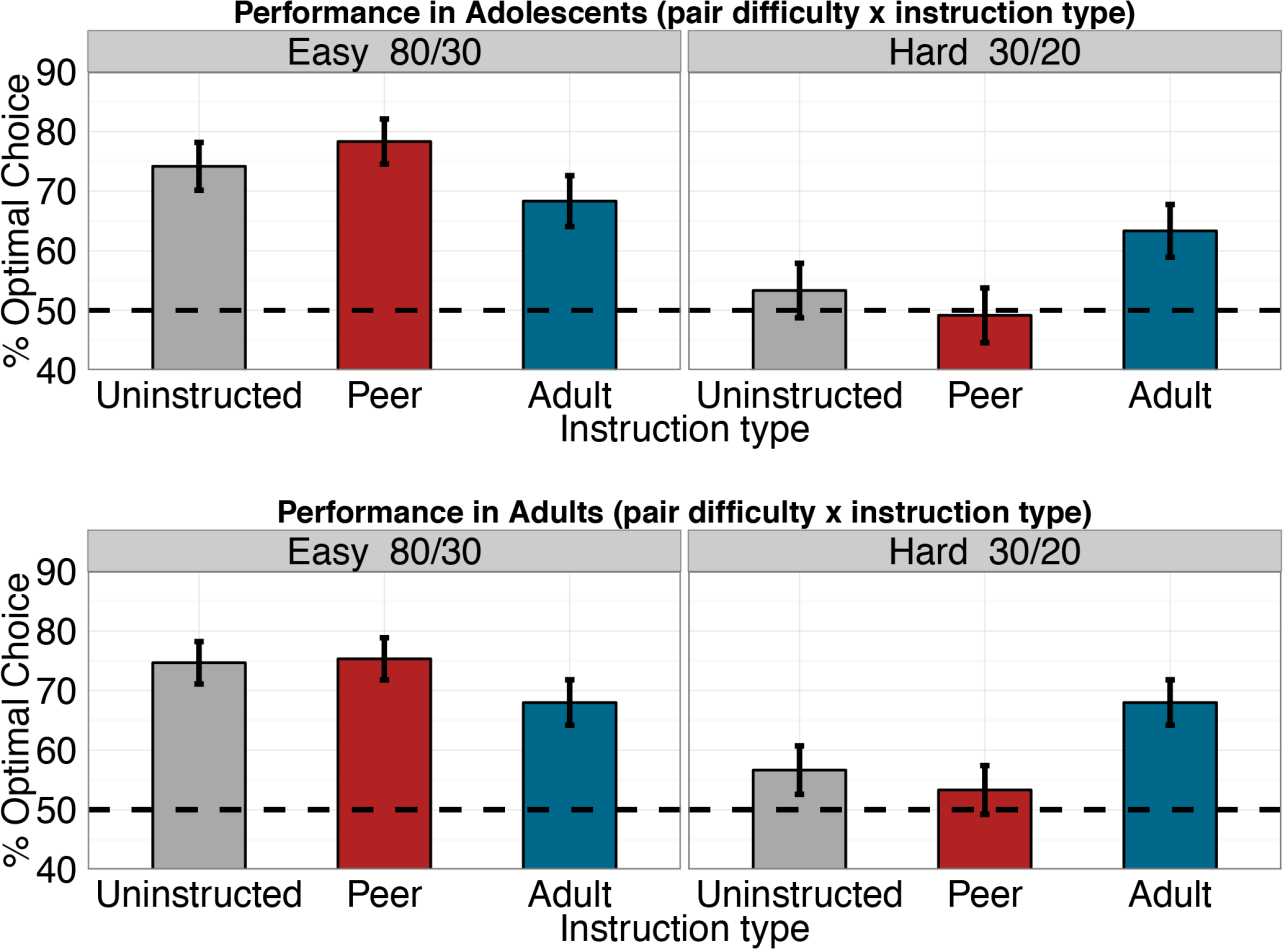
Test phase performance dependence on pair difficulty and instruction type. Harder pairs (30/20%) led to significantly lower performance than easier ones (80/30%). Pair difficulty and instruction type also had an interactive effect on performance. Adult instruction impaired performance for the easy pair, and improved performance for the hard pair, compared to the uninstructed pairs. In contrast, peer instruction improved performance for the easy pair and impaired performance for the hard pair, compared to the uninstructed pairs. None of the effects differed between age groups. Error bars represent SEM.

Finally, we used a generalized linear model to test the effects of instruction type (uninstructed, peer, and older-adult), pair difficulty (easy: 80:30 and hard: 30:20), and age group (adolescent and adult) on performance, indexed as choosing the higher valued option. This analysis included three easy pairs (AD 80:30 uninstructed, AF 80:30 peer instructed, and AH 80:30 adult instructed) and three difficult pairs (DB 30:20 uninstructed, FB 30 peer-instructed:20, and HB 30 adult-instructed:20), which represent the components of the two instruction bias scores. Performance did not differ by instruction type (p = 0.61) or age group (p = 0.79), but did differ as a function difficulty (*X* ^2^ = 7.12, df = 1, p = 0.008). Specifically, performance was lower for harder pairs (30:20) than easier pairs (80:30) (log-odds difference = 1.23, CI (0.39, 2.09), p = 0.01; Fig 6), regardless of instruction type. However, there was also a difficulty-by-instruction type interaction (*X* ^2^ = 10.02, df = 2, p = 0.007). Performance was enhanced by the older adult instruction for the harder pair (30 adult instructed:20) and diminished for the easier pair (80:30 adult instructed) relative to the uninstructed pairs (log-odds difference = -1.25, CI (-2.07, -0.48), p = 0.02). This interaction effect reflects the same biasing influence of older adult advice on test phase choices observed in our analysis of the instruction bias score. In contrast, the opposite was seen for the peer instruction (log-odds difference = 1.04, CI (0.37, 1.9), p = 0.01) for which there was a tendency to go against peer advice. There were no interaction effects of age-group (all p-values > 0.6).

### Effects of Estimated IQ

Performance during the learning phase was positively correlated with IQ (r = 0.37, p = 0.02), with similar patterns in adolescents (r = 0.37, p = 0.12) and adults (r = 0.35, p = 0.13). When examined by pair, there was a marginal correlation between IQ and performance for the harder uninstructed pair (CD; r = 0.31, p = 0.058), but not for the other pairs (AB 80:20, p = 0.5; EF 70:30 peer, p = 0.22; GH 70:30 older adult, p = 0.11). Similarly, test phase performance was marginally correlated with IQ (r = 0.31, p = 0.058), but IQ was not associated with instruction biases (peer bias: r = 0.11, p = 0.49; older adult bias: r = -0.08, p = 0.61).

### Extent of Identification with Instruction Source

Participants were asked at the end of the experiment to estimate the age of each advisor. All participants perceived the adult advisor to be older than the peer advisor (mean age difference = 20.3 (SD = 11.1) t = 10.4, p<0.0001), and adolescents perceived a greater age difference between the sources than adults did (adolescents: 26.0 (SD = 11.5); adults: 16.4 (SD = 9.2); t = 2.50, p = 0.020). Adolescents and adults had similar estimates for the peer’s age relative to their own (adolescents: 2.9 years older (SD = 4.0); adults: 3.4 years older (SD = 7.1); t = -0.22, p = 0.82), but varied more on estimates of the older adult’s age difference (adolescents: 28.9 years older (SD = 12.6); adults 20.3 years older (SD = 10.8); t = 2.05, p = 0.052).

We tested whether adolescents identified more with the peer versus the older adult using the Inclusion of Other in Self scale [39,40], a 7-picture scale of two increasingly overlapping circles labeled “Self” and “Other”. We calculated a difference score between participants’ “relatedness” (IOS score of 1–7) with the peer and the older adult (IOS Peer—IOS Adult). This difference score was negatively correlated with age (r = -0.34, p = 0.023), suggesting that adolescents identified more with the peer advisor than the older adult advisor. In contrast, adults did not identify more with one advisor than another. Learning phase performance did not correlate with peer IOS (r = -0.1, p = 0.5) or adult IOS (r = -0.14, p = 0.36). However, test phase performance negatively correlated with peer IOS (r = -0.35, p = 0.02) and adult IOS (r = -0.38, p = 0.01), suggesting that those who identified the least with the peer and older adult advisors had the highest performance across all pairs. Peer IOS did not predict peer bias (r = 0.15, t(43) = 0.98, p = 0.36), however higher adult IOS was associated with higher adult bias (r = 0.41, t(43) = 2.95, p = 0.005), suggesting that participants were more influenced by the older adult advice to the extent that they identified with the older advisor.

### Participant Awareness of Bias

To determine each age group’s basic tendency to follow instruction, we examined the first trials in the learning phase in which an instructed stimulus was presented, before a participant received any feedback. We examined this initial choice using a general linear model that used ‘choosing to follow advice’ as the dependent measure, and age group, source of advice, and their interaction as independent predictors. There was no significant effect of pair, age group, or their interaction (all p-values > 0.24), with only the intercept being significant (p = 0.033). 74% of participants followed advice on the first instance of each instructed pair—75% and 70% of adolescents followed peer and older advice, respectively, and 68% and 84% of adults followed peer and older adult advice. These results suggest that participants in both age groups followed advice initially, and showed no systematic preference for either source of advice.

Upon finishing the task, forty of the 45 participants answered three questions about the advice they received: 1) Whose advice they thought was more helpful; 2) Whose advice they followed more; and 3) If they began to doubt the advice. Based on chi-square tests there were no age group differences for any of these questions (all p-values > 0.32). Further, neither advice source was considered to be more helpful (p = 0.72), and the majority reported following neither (n = 13) or both (n = 17) sources of advice (*X* ^2^ = 11.6, p = 0.009). Most strikingly, 35 out of the 40 participants reported doubting both sources of advice (*X* ^2^ = 83.4, p < 0.0001). These data indicate the participants’ clear recognition that the advice they received was inaccurate, and yet they still showed an older-adult instruction bias. This suggests that the older adult instruction bias is unlikely to stem from explicit knowledge.

## Discussion

The current study examined whether peer advice biases adolescent behavior using a task in which participants received inaccurate advice from a gender-matched peer and older adult. Previous studies examining the influence of instruction without a clear social source have observed an instruction bias in adults [29,32,34], whereas children and adolescents exhibited unbiased experiential learning [31]. We predicted that adolescents might be more influenced by peer advice than advice received from older adults. First, our results failed to show an overall bias for either adolescents or adults. However, when considering the source, both age groups showed evidence of being biased by the older adult’s advice and showed no evidence of being biased by the peer advice. These results underscore that older adults can guide behavior even in the face of contradictory experience, and highlight the absence of peer influence on adolescent behavior in certain situations.

This study and others of similar task design have generally used inaccurate advice to elicit an instruction bias [28,29,31,34]. When experiential learning alone can approach near ceiling levels, as has been observed in children in a task similar to ours [5], accurate advice is unlikely to yield further improved performance. In a task of greater difficulty, in which participants’ experiential learning did not approach ceiling levels, accurate advice resulted in better performance than that achieved through experiential learning alone [41]. This is consistent with the theory that advice, whether accurate or inaccurate, augments the value of a recommended stimulus relative to an equally valued uninstructed stimulus. While our use of inaccurate advice in our study was necessary to clearly dissociate advice from experience, we expect that accurate advice in an appropriately designed task would yield a similar differential influence of source.

Choice behavior typically reflects the integration of one’s experiences with external information. This strategy is usually beneficial because following other’s advice can often improve one’s performance in new situations [42]. Both accurate and inaccurate advice have been found to similarly bias subsequent behavior [28,29,41,43], with the influence of advice varying as a function of genetic [32], psychiatric [33], and developmental [31] factors. The perceived quality of advice appears to influence how strongly it is followed [44], with individuals preferring advisors that appear more credible [45,46]. Expert advice has been shown to be more highly valued than novice advice [47], and is less devalued when found to be incorrect [48]. Thus, relying on advice from experts may provide a cognitive shortcut by “offloading” the evaluations that inform a decision [49]. Adolescents may perceive peers to have expertise in social domains, but may find their advice less credible in non-social contexts. Older adults, on the other hand, may be considered to have more general expertise, leading participants to be more influenced by their advice when making their decisions in our task. However, as we did not ask participants to rate the credibility of the advisors, it is unclear whether the differential biasing effects observed in our study depended on perceptions of advisors’ expertise.

In this study we chose to make several potentially confounding factors associated with the source of information fixed (e.g. gender, age, familiarity). This design failed to reveal the frequently observed adolescent sensitivity to peers [14,23,50,51] that we hypothesized would be reflected in a peer bias. However, both advisors were strangers to the participants, and perhaps a degree of affiliation is required for the peer to remain influential. Using familiar peers or older adults might have changed the perception of advice and thereby its influence. However, using the IOS assessment of relatedness, we found that adolescents perceived greater similarity with the peer advisor than the older adult advisor. Adults identified equivalently with the two advisors, potentially due to a smaller perceived age gap between advisors. Despite this difference in perceived relatedness, both adolescents and adults were biased only by the older adult advice. These results suggest that source familiarity does not account for the differential biasing effects observed here, but it is possible that truly familiar advisors might have exerted greater influence on participant behavior.

Although participants exhibited an older adult bias, they were unaware of this tendency. Prior to any potential experiential learning, both age groups followed the peer and older adult advice, suggesting there was no initial preference given to the older source. Similarly, participants showed no sign of favoring the peer or older adult when questioned about the usefulness of the advice they received. Nearly all participants reported doubting both the peer and older adult advice by the end of the task. This suggests that participants had no explicit beliefs that the older adult advice was better, and rather that the instruction bias may stem from implicit mechanisms. This comports with prior theoretical models proposing that instruction bias results from the perturbation of normal feedback processing during the learning phase [29,31]. Despite the participants’ lack of explicit belief in the credibility of either source of advice, instruction bias here was observed only for the older adult advice. This selective influence may stem from an implicit prioritization of older adult advice in our task.

Our results differ in part from previous instructed learning studies [29,31,34] in that we do not find an overall instruction bias, even in adults. This finding may be due to the complexity and cognitive load of the task, as participants had to integrate information from four pairs and two false instructions, rather than three pairs, with one falsely instructed, as has been typically used in this task design [28,29,31]. Instruction is proposed to exert top-down cognitive control over the experiential learning process, biasing learned values [29]. The added load in this study may have diminished the biasing effect typically seen in adults. This may have rendered adult performance more similar to that observed in younger participants, who exhibited minimal instruction bias both here and in a previous study [31]. This interpretation is supported by the correlation between learning and IQ, and by overall poorer performance in the current study compared to previous studies with fewer pairs [5]. Interestingly, the worst performance was observed for the hardest uninstructed pair, for which there was no external information to guide choices. As participants initially followed advice and subsequently realized from experience that it was not beneficial, it is possible that instructions had the unexpected effect of facilitating learning for the instructed pairs. In future work, if a between-subject study design were used in which participants got either peer advice or older adult advice, we would expect cognitive load to be reduced, potentially yielding stronger instruction biases in adults.

In sum, contrary to conventional wisdom that adolescents are disproportionally influenced by their peer group [52], here we show that there are situations in which adolescent behavior seems to be less sensitive to peers. Both adolescents and adults initially followed advice from both sources. However, after experiencing contradictory feedback, both groups failed to be influenced by the peer advice whereas they continued to be biased by the older adult advice. This differential influence potentially reflects an implicit tendency to find older adults more credible in certain choice domains, without any explicit awareness of valuing peer advice less. These results highlight the important role that adults, such as parents and mentors, may play in shaping adolescent decisions, even when their advice runs counter to adolescents’ experience.

## Supporting Information

S1 Table Participant Covariates. A table of covariates, including: age, gender, CARE, IQ, IOS, RPI, and responses to debriefing questions.
(CSV)

S2 Table Learning Phase Choices. A table of participants’ choices made during the learning phase.
(CSV)

S3 Table Test Phase Choices. A table of participants’ choices made during the test phase.
(CSV)
